# Molecular classification and immunologic characteristics of immunoreactive high‐grade serous ovarian cancer

**DOI:** 10.1111/jcmm.15441

**Published:** 2020-06-17

**Authors:** Zheran Liu, Haifang Wu, Jiachen Deng, Haoqing Wang, Zixuan Wang, Ailin Yang, Bowen Liang, Ji Luo, Jianyong Li, Yanmei Xu, Xiaoli Tang, Fen Fu, Libin Deng

**Affiliations:** ^1^ The Second Affiliated Hospital of Nanchang University Nanchang China; ^2^ Department of Biotherapy Cancer Center West China Hospital Sichuan University Chengdu China; ^3^ School of Information Engineering Nanchang University Nanchang China; ^4^ Jiangxi Provincial Key Laboratory of Preventive Medicine School of Public Health Nanchang University Nanchang China; ^5^ School of Basic Medical Science Nanchang University Nanchang China

**Keywords:** high‐grade serous ovarian cancer, immune checkpoints, immune microenvironment

## Abstract

High‐grade serous ovarian cancer (HGS‐OvCa) is one of the most lethal gynaecological malignancies. Molecular classification identified an immunoreactive subtype of HGS‐OvCa; however, the immunologic characteristics of immunoreactive HGS‐OvcA remain unclear. In this study, 121 immunoreactive HGS‐OvCa samples were identified from a meta‐analysis of 5 large transcriptome profiling data sets using a cross‐platform immunoreactive HGS‐OvCa subgroup‐specific classifier**.** By comparing the gene expression profiles of immunoreactive HGS‐OvCa samples and normal tissues, 653 differentially expressed genes (DEGs) were identified. KEGG pathway analysis revealed that the leukocyte transendothelial migration pathways were significantly enriched in the immunoreactive HGS‐OvCa. Protein‐protein interaction analysis identified a module that showed strong involvement of the immune‐related chemokine signalling pathway. Moreover, the GSEA enrichment analysis showed a T‐cell subgroup and M1 macrophages were significantly enriched in immunoreactive OvCa compared with normal samples. Macrophage infiltration levels were significantly elevated in immunoreactive HGS‐OvCa compared with other OvCa subtypes. In addition, expression of immune checkpoint molecules *VTCN1* and *IDO1* was significantly increased in immunoreactive HGS‐OvCa. In summary**,** our results suggest that the immunoreactive HGS‐OvCa has unique molecular characteristics and a tumour‐associated immune microenvironment featured by increased infiltration of macrophages, rather than lymphocytes. *VTCN1* could be potential targets for the treatment of immunoreactive HGS‐OvCa.

## 1 BACKGROUND

High‐grade serous ovarian cancer (HGS‐OvCa) is one of the most common malignancies of the female reproductive system, ranking third in morbidity among all gynaecological cancers.^1^ Although surgery combined with chemotherapy is used as a standard of care, 75% of treated patients may experience drug resistance, relapse and short survival times. Only a fraction of the treatment‐sensitive patients have a long disease‐free survival.^2^

The clinical application of immune checkpoint inhibitors is one of the great successes of anticancer treatment in recent years.^3^ The immune checkpoints mediate the balance between immune surveillance and immune escape.^4^ Immune checkpoint inhibitors such as PD‐1/PD‐L1 inhibitors have shown promising antitumour activity and limited adverse effects in treating several types of cancers.^5^ Although HGS‐OvCa had a strong immune recognition susceptibility,^6^ the response of HGS‐OvCa to anti‐PD‐1/PD‐L1 monotherapy was minimal in some patients. The lack of therapeutic efficacy may be because of the insufficient and heterogeneous expression of PD‐1 in the tumour‐associated microenvironment of HGS‐OvCa.^7^ However, there still lacks an effective way to distinguish the immunotherapy‐sensitive cohort in HGS‐OvCa patients.

Cancer heterogeneity is directly related to disease progression and patient prognosis. Take breast cancer as an example—it was classified into five main subtypes: luminal A, luminal B, HER2‐overexpression, basal‐like and normal‐like. The prognosis of HER2‐positive breast cancer patients who use trastuzumab as a targeted treatment is significantly better than that of patients with basal‐like breast cancers.^8^ HGS‐OvCa also exhibits high heterogeneity among its molecular characteristics. Similar to breast cancer, the classifications of different subtypes of HGS‐OvCa might be useful to predict the drug sensitivity and the treatment outcome of patients.^9^

The application of high‐throughput gene expression profiling methods enables accurate identification of the molecular subtypes of HGS‐OvCa. Tothill et al^10^ identified four distinct molecular subtypes of HGS‐OvCa. Among them, C1 (mesenchymal) subtype correlates with the high stromal response, the C2 (immunoreactive) subgroup exhibits high expression of immune cell‐related genes, the C4 (differentiated) subtype shares some common features with serous borderline tumours, and the C5 (proliferative) subtype demonstrates low expression levels of differentiation markers. The Cancer Genome Atlas (TCGA) Research Network showed that immunoreactive HGS‐OvCa exhibits high expression levels of the T‐cell chemokine ligands CXCL11 and CXCL10, and its receptor CXCR3. They suggested that the C2 subset has a unique immune microenvironment. Thus, this subset may benefit from immune checkpoint‐targeted treatments.

However, controversy exists on whether the prognosis of patients could be affected by the molecular subtypes of HGS‐OvCa. Zhang et al^11^ classified the survival pattern of four subtypes of HGS‐OvCa using the data from TCGA. They concluded that it was the tumour‐associated stroma, not HGS‐OvCa subtypes, that was associated with the patient's prognosis.^11^ On the other hand, Shilpi et al^12^ developed a novel classification system using the exon array and RNA sequencing data of HGS‐OvCa from TCGA. They showed that the molecular subtypes of HGS‐OvCa could stratify patients into different survival patterns.^12^ More importantly, they revealed that the prognosis of the immunoreactive subtype was not the best, which was unexpected.^12^ Meanwhile, Fucikova et al^13^ emphasized that high expression levels of immune checkpoint molecules PD‐L1 and TIM‐3 were strong prognostic factors of HGS‐OvCa. However, the prognostic impact of immune checkpoint molecules on each subtype of HGS‐OvCa patients was not determined in previous studies.

The aim of the present study was to elucidate the unique molecular and immune characteristics of the immunoreactive HGS‐OvCa and to identify the potential immune checkpoint inhibitors for its treatment. Here, we established a cross‐platform classifier to distinguish the immunoreactive subtypes based on gene expression profiles. We identified the differentially expressed genes (DEGs) between immunoreactive HGS‐OvCa tissues and normal tissues according to the classifier. A series of bioinformatic analyses were conducted to investigate the distinct molecular characteristics of immunoreactive HGS‐OvCa. After that, we compared the enrichment status of immune cells between immunoreactive OvCa tissue and the normal tissue. Then, the comparison of the immune cell abundance and fractions between subtypes was also performed. The immune checkpoints expression patterns in subgroups were evaluated and validated by multiple methods using varies datasets.^14^ Finally, we further explored the possible mechanism that maintains the immune‐balanced microenvironment in immunoreactive HGS‐OvCa.

## 2 MATERIALS AND METHODS

### 2.1 Establishment of a cross‐platform classifier of immunoreactive HGS‐OvCa

The mRNA profile of ovarian cancer and related data (ovarian cancer, RNA Seq V2, Illumina GA‐DNASeq) in the TCGA database was acquired from the UCSC cancer genome database (http://xena.ucsc.edu/). The expression data of genes shared across multiple data sets including the TCGA dataset and 5 Gene Expression Omnibus (GEO) datasets (GSE06008, GSE18520, GSE26712, GSE27651, GSE9891) were extracted (Table 1). These gene expression values were normalized and scaled using the scikit‐learn library in R. The 299 HGS‐OvCa samples from TCGA were sorted into four subtypes, including differential, immunoreactive, proliferative and mesenchymal types, according to TCGA classification (Additional file S1).^15^ The top 50 expressed genes were selected as feature genes based on the filter methods for feature selection^16^ and an expression matrix was formed. Then, the 299 well‐characterized samples from the TCGA mRNA expression data set were randomly divided into a 250‐case training cohort and a 49‐case validation cohort, respectively. Samples in the training cohort were classified as either the immunoreactive type or non‐immunoreactive type by applying cluster analysis using the BP neural network model in Python. The same method was applied to the validation cohort to verify the accuracy of the model.

**TABLE 1:** The characteristics of selected GSE datasets

Dataset	Total samples amounts	Selected samples amounts	Sample amounts of the immunoreactive subtype	Case/Control	Country	PMID	Platforms	Gene#	Gene Chip
GSE6008	103	103	19	99/4	USA	16452189/19843521/17418409/27538791	GPL96	14378	HG‐U133A
GSE18520	63	63	17	53/10	USA	19962670	GPL570	23882	HG‐U133_Plus_2
GSE26712	195	195	27	185/10	USA	18593951/25944803	GPL96	14378	HG‐U133A
GSE27651	49	41	8	35/6	USA	21451362	GPL570	23882	HG‐U133_Plus_2
GSE9891	285	248	50	214/34	AUS	18698038	GPL570	23872	HG‐U133‐Plus‐2
Total	695	650	121	586/64					

The model accuracy was optimized and computed by applying the set model to GSE9891 as external validation. The results were compared with the findings of Tothill.^10^ A receiver operating characteristic (ROC) curve was constructed to identify the accuracy of the model by computing the area under the curve (AUC). Eventually, a cross‐platform model was established and applied to the other four GEO datasets to classify them into immunoreactive and non‐immunoreactive HGS‐OvCa.

### 2.2 A meta‐analysis based on the cross‐platform model

GEO2R (http://www.ncbi.nlm.nih.gov/geo/geo2r) were used to computed the *P*‐value, adjust. *P* value, *t* value, LogFC value and *B* value of each sequencing site of the corresponding probe. For the same gene, the most significant sequencing site remains and others were excluded. Then, the commonly expressed genes among the four GEO data sets were obtained using Perl and the Merge package in R software. Two meta‐analyses were performed on four GSE data sets, including cancer tissues, and the control group, using the MAMA and RankProd package. Z scores (which had |7| as a cut‐off value) and the pval‐test (which had |5| as a cut‐off value) were used to filter the DEGs. Genes that met the above criteria were regarded as the final selected DEGs.

### 2.3 GO annotations and KEGG pathway enrichment analysis

The Gene Ontology (GO)^17^ and Kyoto Encyclopedia of Genes and Genomes (KEGG) pathway^18^ are two powerful databases to explore the underlying activated or inhabited pathways in cancers. KEGG and GO pathway analyses were performed on DEGs in immunoreactive HGS‐OvCa with a false discovery rate (FDR) of less than 0.1 using the WEB‐based GEne SeT AnaLysis Toolkit (http://www.webgestalt.org/option.php).^19^

### 2.4 Construction of the protein‐protein interaction (PPI) network

To obtain a system‐level understanding of the cellular function and biological activity in immunoreactive HGS‐OvCa, we analysed the DEGs with the Search Tool for the Retrieval of Interacting Genes (STRING, http://string‐db.org) database.^20^ PPI networks were constructed with confidence scores greater than 0.4 as the significance cut‐off value. The acquired data were visualized using Cytoscape software.^21^

### 2.5 Identification of hub genes and significant modules

Hub genes were identified based on eigenvector centrality using CentiScaPe 2.1.^22^ Eigenvector centrality is a measurement to evaluate the influence of a node in a certain network. The most significant cluster gene module was chosen with the condition that the degree cut‐off = 2, node score cut‐off = 0.2, k‐core = 2, and max. depth = 100 using Molecular Complex Detection (MCODE) software. Moreover, the DEGs in each module with an FDR less than 0.1 were subjected to the WEB‐based GEne SeT AnaLysis Toolkit to perform GO and KEGG analyses.

### 2.6 Kaplan‐Meier (KM) survival analysis

KM plotter (http://kmplot.com/analysis/) is an effective online program that contains 1816 HGS‐OvCa patients.^23^ The gene expression file of the hub genes and relative survival information was extracted from the GEO, European Genome‐phenome Archive (EGA) and TCGA databases. Survival analysis was performed to explore the relationships between the expression levels of selected genes and the prognosis of patients with HGS‐OvCa.

### 2.7 Analysis of enrichment and abundance of tumour‐infiltrating immune cells in immunoreactive HGS‐OvCa

Gene Set Enrichment Analysis (GSEA) was performed to evaluate the differential expression between immunoreactive HGS‐OvCa and normal tissue based on a pre‐defined leukocyte gene signature matrix using the WEB‐based GEne SeT AnaLysis Toolkit.^24^

To investigate the immune cell abundance in immunoreactive HGS‐OvCa, we used the Tumor Immune Estimation Resource (TIMER; cistrome.shinyapps.io/timer) to estimate the abundance of immune cells (B cells, CD4 T cells, CD8 T cells, macrophages and dendritic cells) in TCGA samples. The abundance data were analysed and verified using pathological estimations.^25^

### 2.8 Characterization of the expression patterns of the selected genes in bulk expression and single‐cell RNA‐sequencing (scRNA‐seq) data sets

The expression levels of specific genes in four subtypes of HGS‐OvCa were acquired from 5 GSE data sets. The comparison between subtypes was applied by using the beanplot package in R 3.5.1.

The raw data of scRNA‐seq of HGS‐OvCa were obtained from Shih et al^26^ (GSE118828). Seurat, another R package, was used to analyse the scRNA‐seq data. The cell population that expressed the selected genes were identified after data normalization, scaling, linear dimensional reduction and visualization using UMAP.^27^

## 3 RESULTS

### 3.1 Establishment of a cross‐platform classifier of HGS‐OvCa subtypes

**FIGURE 1:**
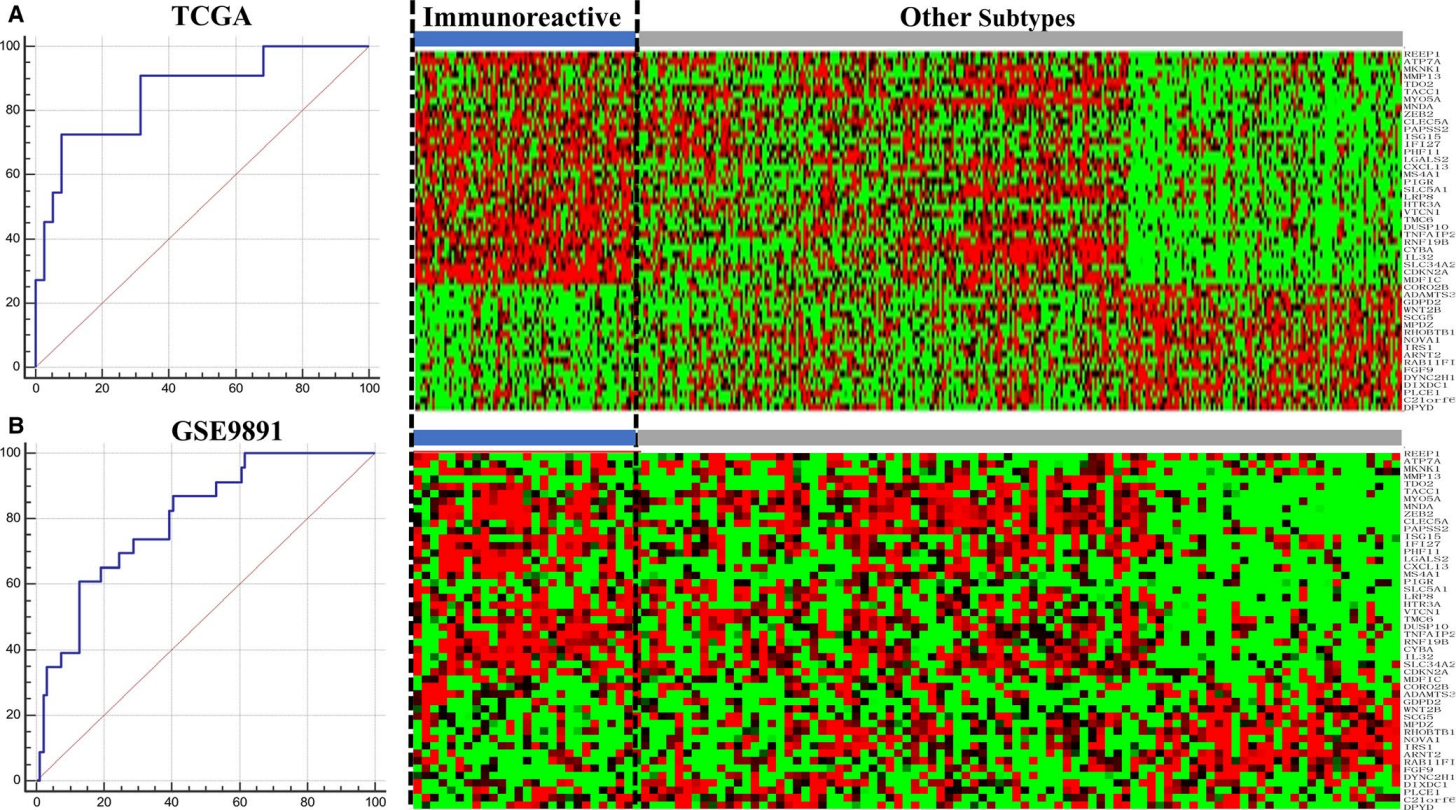
The construction and validation of the OvCa classifier. A, The accuracy when the classifier is applied to samples in TCGA validate cohort (Left). The heatmap of the expression status of the classifier‐adopted feature genes in the TCGA cohort (Right). B, The accuracy when the classifier is applied to samples in the GSE9891 validate cohort (Left). The heatmap of the expression status of the classifier‐adopted feature genes in GSE9891 cohort (Right)

To build a comprehensive cross‐platform classifier, we first extracted 10 411 commonly shared genes after screening the HGS‐OvCa mRNA expression profiling data in 5 GEO data sets (650 HGS‐OvCa samples) and the TCGA (299 HGS‐OvCa samples) data set. Then, 299 well‐characterized samples from the TCGA data set were randomly divided into a 250‐case training cohort and a 49‐case validation cohort, respectively. Samples in the training cohort were classified as the immunoreactive type or non‐immunoreactive type by applying BP neural network model using the selected 50 feature genes. The accuracy of the model applied to the TCGA validation cohort was 85.6%, with a 95% confidential interval (CI) of 0.727‐0.940 (Figure 1A). The same method was applied to the external validation cohort to verify the accuracy of the model by applying the set model to GSE9891 and comparing the results with those of Tothill et al.^10^ The ROC curve suggested that the accuracy of the model applied to GSE98981 was 80.2%, with a 95% CI of 0.718‐0.870 (Figure 1B).

The expression profiles of marker genes selected by TCGA^15^ further confirmed the accuracy of our classifier. As shown in Additional file S2, the expression levels of these genes among the different HGS‐OvCa subtypes were consistent with the original conclusion. For example, CXCL11 and CXCR3 were highly expressed in immunoreactive HGS‐OvCa, while the expression of CXCL12 was low.

### 3.2 Exploration of the molecular characteristics of immunoreactive HGS‐OvCa

**FIGURE 2:**
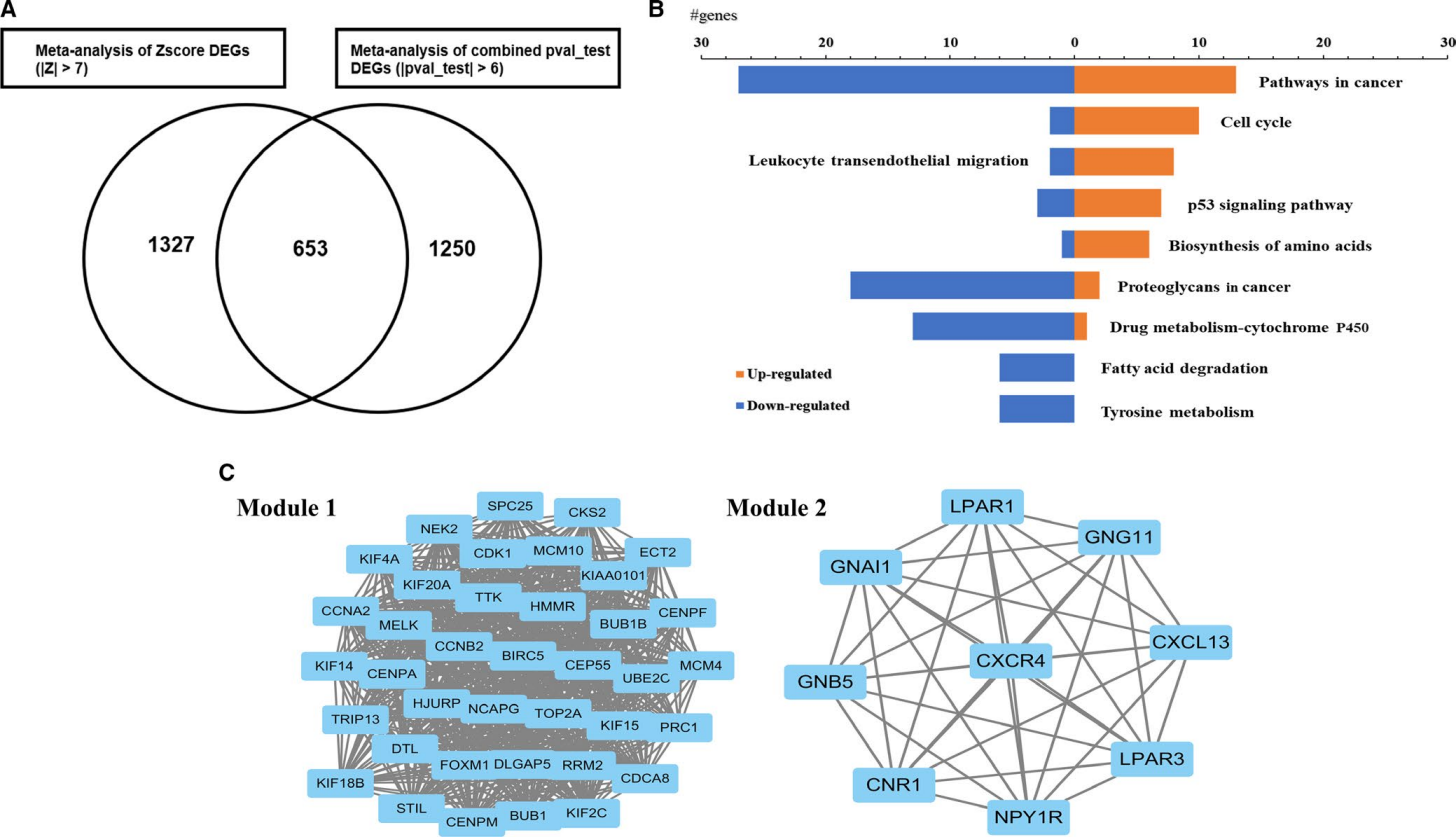
The characteristics and enrichment status of DEGs identified from immunoreactive HGS‐OvCa. A, The 653 overlapped DEGs selected based on the standard of | pval_test |> 6 and | Z |> 7 using Venny 2.1.0. B, The visualization of KEGG analysis for up‐regulated DEGs and down‐regulated DEGs in immunoreactive HGS‐OvCa. C, The top 2 most significant modules in the PPI network. DEGs were related to the cell cycle pathway in Module 1 and were related to the chemokine signalling pathway in Module 2

We compared the expression levels of 12,818 genes in the 5 GEO data sets. Under the condition of |pval_test| > 5 and |Z‐score| > 7, we identified 653 DEGs (Figure 2A), including 226 up‐regulated genes (Additional file S3) and 427 down‐regulated genes (Additional file S4) between immunoreactive and normal ovarian surface epithelium tissues. Some DEGs overlapped with the featured genes selected by the BP neural network.

To explore the molecular functio

ns of the DEGs, we used the WEB‐based GEne SeT AnaLysis Toolkit to perform pathway enrichment analyses according to KEGG and GO annotation. The KEGG pathway analysis results showed that up‐regulated DEGs were mostly enriched in cell cycle and leukocyte transendothelial migration (TEM) pathways (FDR < 0.1, Figure 2B, Additional fileS5). GO biological process analysis also indicated that 65 up‐regulated DEGs were enriched in cell cycle pathways (Additional file S6). In our previous work,^28^ we concluded that the most significant up‐regulate‐gene enrichment pathway between ovarian cancer tissues and normal ovarian epithelium tissues was also the cell cycle pathway. This result confirmed that aberrant functions of the cell cycle still are the primary molecular characteristic in immunoreactive HGS‐OvCa, which is the same as the general HGS‐OvCa characteristics.

A PPI network that included 509 nodes and 2542 edges was built with the STRING database (the confidence score was higher than 0.4). We screened 10 hub genes (*PRKCA, FGF13, MYH14, UBE2I, MELK, AKT3, FAS, KDR, PAX8* and *CXCR4*) based on eigenvector centrality. Additionally, the top 2 significant modules were acquired from the PPI network using MCODE (Figure 2C). Functional annotation indicated that Module 1 was associated with the cell cycle, which is consistent with the molecular features of general HGS‐OvCa.^28^ Module 2 is a distinctive module in immunoreactive HGS‐OvCa and is related to the chemokine signalling pathway (*P* = 1.32 × 10^−6^, Additional file S7) according to KEGG analysis. The chemokine signalling pathway is responsible for attracting leukocytes or other immune cells to the inflammatory location to activate the immune response. It is essential for leukocyte TEM process, which was found activated in the immunoreactive HGS‐OvCa. In conclusion, the results of KEGG analysis in DEGs and module genes showed that immunoreactive HGS‐OvCa has distinct immune‐related molecular characteristics.

**FIGURE 3:**
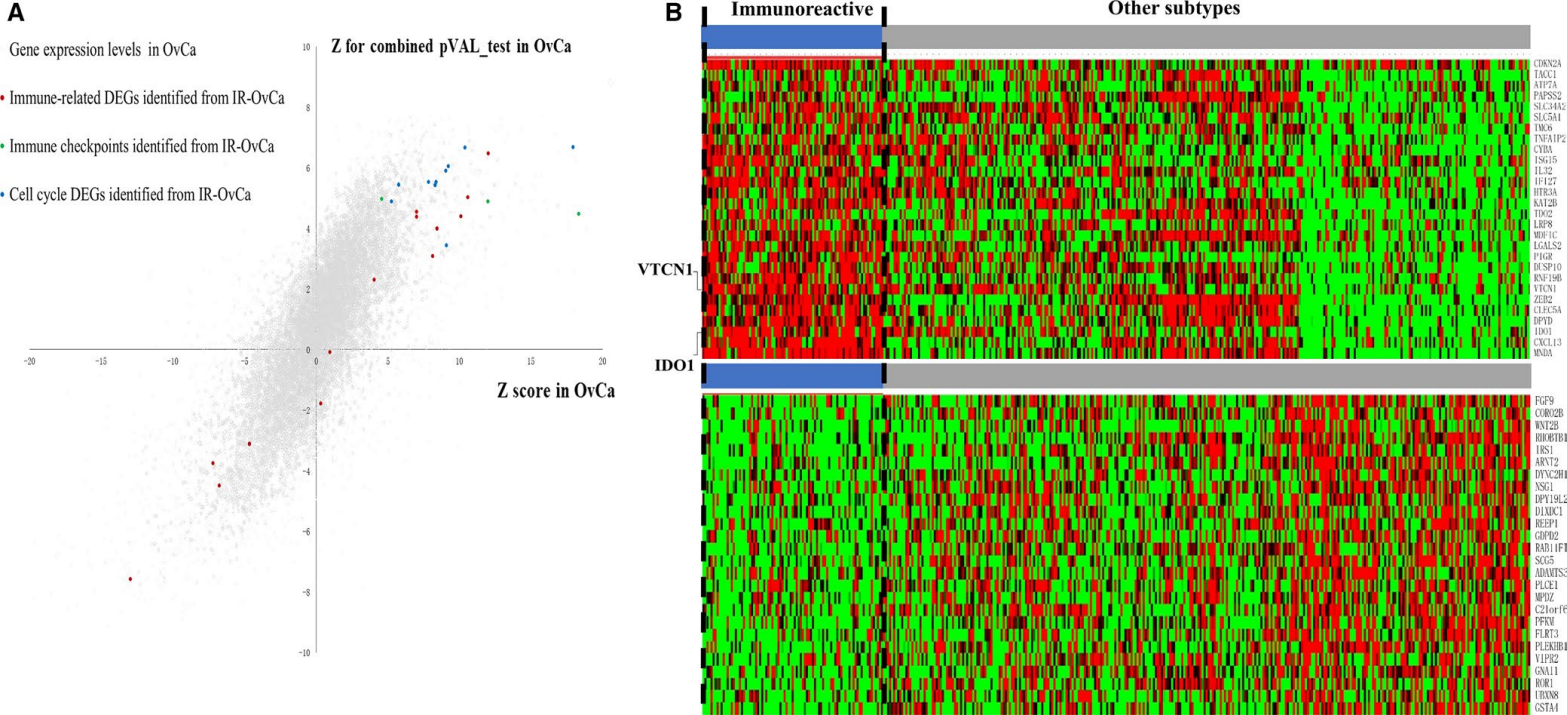
The unique genetic characteristics of immunoreactive HGS‐OvCa compared with the general HGS‐OvCa and other subtypes. A, The scatter diagram of specificity of DEGs in immunoreactive HGS‐OvCa (IR‐OvCa) compared with the general HGS‐OvCa. Both Z for combined pVAL‐test and Z score are proportional to the gene expression level. the grey dots showed the expression status of genes in OvCa according to previous research.^28^ The red, yellow and green dots showed the expression status of the different gene sets (that identified from IR‐OvCa) in general OvCa. B, The heatmap of the 28 significantly up‐regulated gene (FDR < 0.01) in the TCGA HGS‐OvCa cohort between immunoreactive type and non‐immunoreactive type. C, The heatmap of the 26 significantly down‐regulated genes (FDR < 0.01) in the TCGA HGS‐OvCa cohort between immunoreactive subtype and non‐immunoreactive subtype

To further explore the molecular characteristics of immunoreactive HGS‐OvCa, the up‐regulated and down‐regulated DEGs of immunoreactive HGS‐OvCa were compared with the DEGs between tumour and normal control in the general HGS‐OvCa samples.^28^ 104 immunoreactive‐subtype‐specific up‐regulated and 249 immunoreactive‐subtype‐specific down‐regulated DEGs were determined among the 653 immunoreactive HGS‐OvCa DEGs (Additional file S8, Additional file S9). It shows that the up‐regulated cell cycle pathway is commonly shared by both immunoreactive HGS‐OvCa and general HGS‐OvCa. The immune‐related genes found by KEGG and PPI analysis in the immunoreactive HGS‐OvCa were mainly up‐regulated. The expression patterns of these genes in the immunoreactive HGS‐OvCa are different from the general HGS‐OvCa (Figure 3A).

In addition, we re‐calculated the DEGs between immunoreactive type and non‐immunoreactive type based on a cut‐off of FDR < 0.01, 28 significant up‐regulated genes and 26 significant down‐regulated genes were identified (Figure 3B,C). Two immune checkpoint molecules, VTCN1 and IDO1, were found to be distinctly up‐regulated when compared with normal tissue. Both genes are up‐regulated compared with other subgroups of HGS‐OvCa. The Kaplan‐Meier survival analysis showed that three immune checkpoint molecules IDO1, VTCN1 and CX3CL1 were associated with prognosis (Additional file S10).

### 3.3 Investigation of the immune characteristics in immunoreactive HGS‐OvCa

**FIGURE 4:**
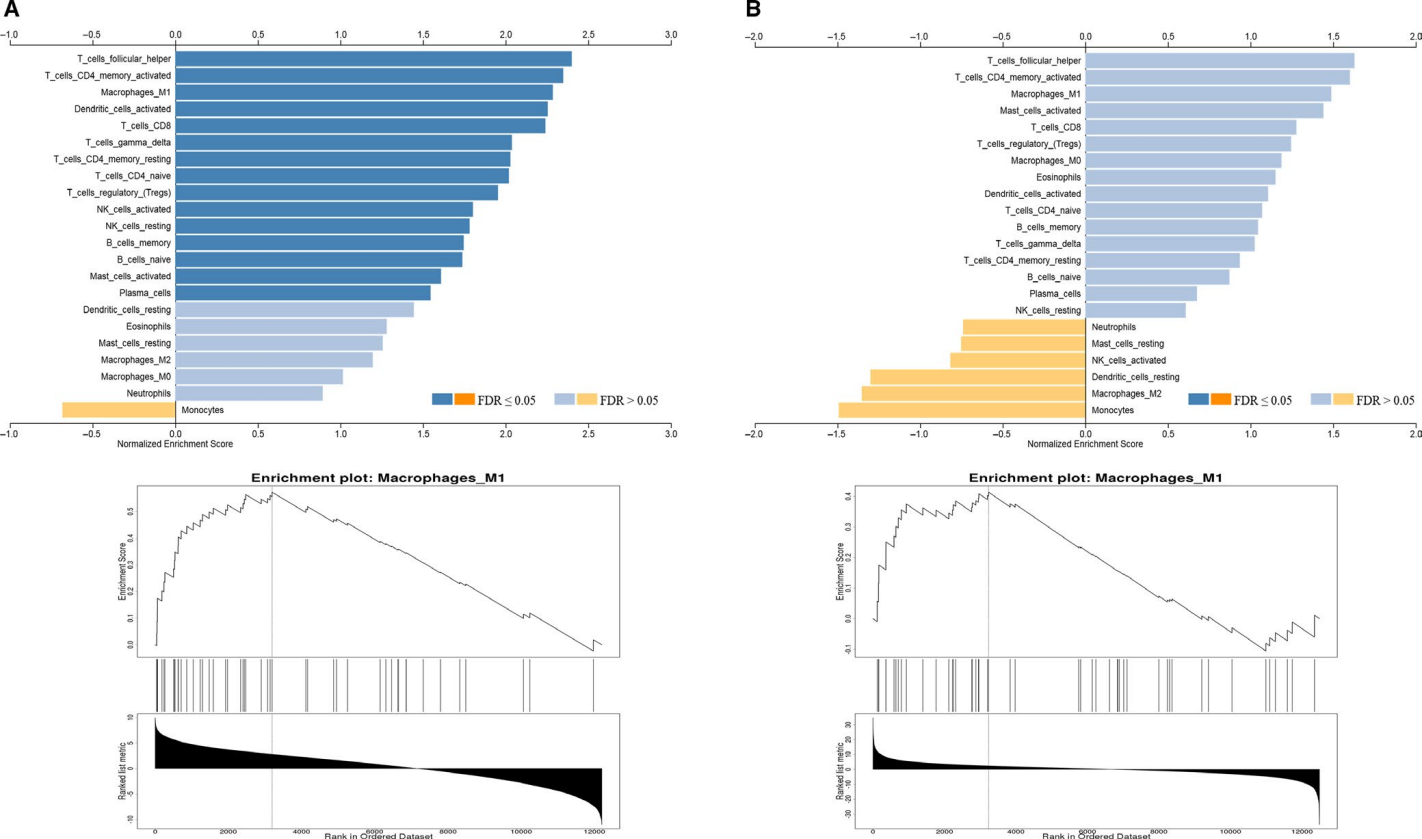
GSEA analysis of the enrichment of DEGs in immune cells. A, based on the results of the Pvalue test. B, based on the results of the Zscore test

To understand the involvement of immune cell subsets in immunoreactive HGS‐OvCa, GSEA was performed to investigate the enrichment of several immune cell subtypes in immunoreactive HGS‐OvCa compared with normal tissues using a leukocyte signature matrix,^24^ which was constructed by applying CIBERSORT (cell‐type identification by estimating relative subsets of RNA transcripts).^24^ The GSEA result shows the immunoreactive HGS‐OvCa DEGs have significantly enriched T‐cell follicular helper cells, CD4 memory T cells and M1 macrophages (Figure 4), activated dendritic cells and CD8 T cells.

**FIGURE 5:**
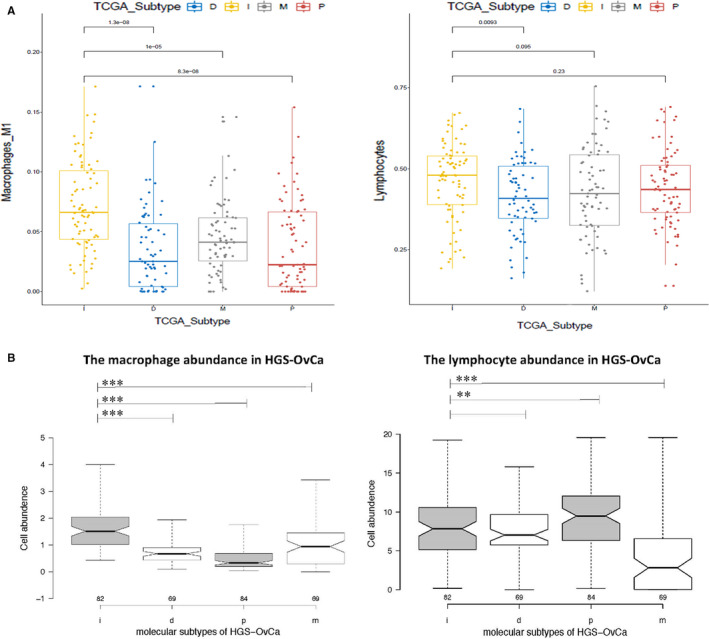
The estimation of cell fractions and abundance in immunoreactive HGS‐OvCa in the TCGA cohort, compared with other subgroups. A, The estimated fractions of M1 macrophages and lymphocytes according to the PanTCGA analysis. It showed the lymphocytes are slightly up‐regulated while M1 macrophages are significantly up‐regulated in immunoreactive HGS‐OvCa. B, The abundance of macrophages and lymphocytes in HGS‐OvCa tumour‐associated microenvironment according to TIMER

After comparing the immune cell enrichment status between immunoreactive HGS‐OvCa and normal tissues, we analysed the relative abundance of 22 immune cells among the four HGS‐OvCa subgroups. The immune cells fractions of each patient in TCGA have been estimated previously.^14^ Compared with the other three subtypes of HGS‐OvCa, the macrophage fractions were significantly up‐regulated, while the lymphocyte fractions showed no statistically significant changes in immunoreactive HGS‐OvCa (Figure 5A). In addition, analysis of the Tumor Immune Estimation Resource (TIMER; cistrome.shinyapps.io/timer) further confirmed that the abundance of macrophages in HGS‐OvCa was higher than other subtypes of immune cells in the tumour‐associated microenvironment, while lymphocyte abundance was not significantly different (Figure 5B). To conclude, the results show that macrophage infiltration, instead of lymphocyte infiltration, maybe one of the key characteristics of immunoreactive HGS‐OvCa. It is worth noting that, different from the GSEA results, the abundance of T cells in immunoreactive HGS‐OvCa was similar to other subtypes of HGS‐OvCa.

### 3.4 The exploration of the relationships of macrophages and VTCN1 expression based on single‐cell analysis

**FIGURE 6:**
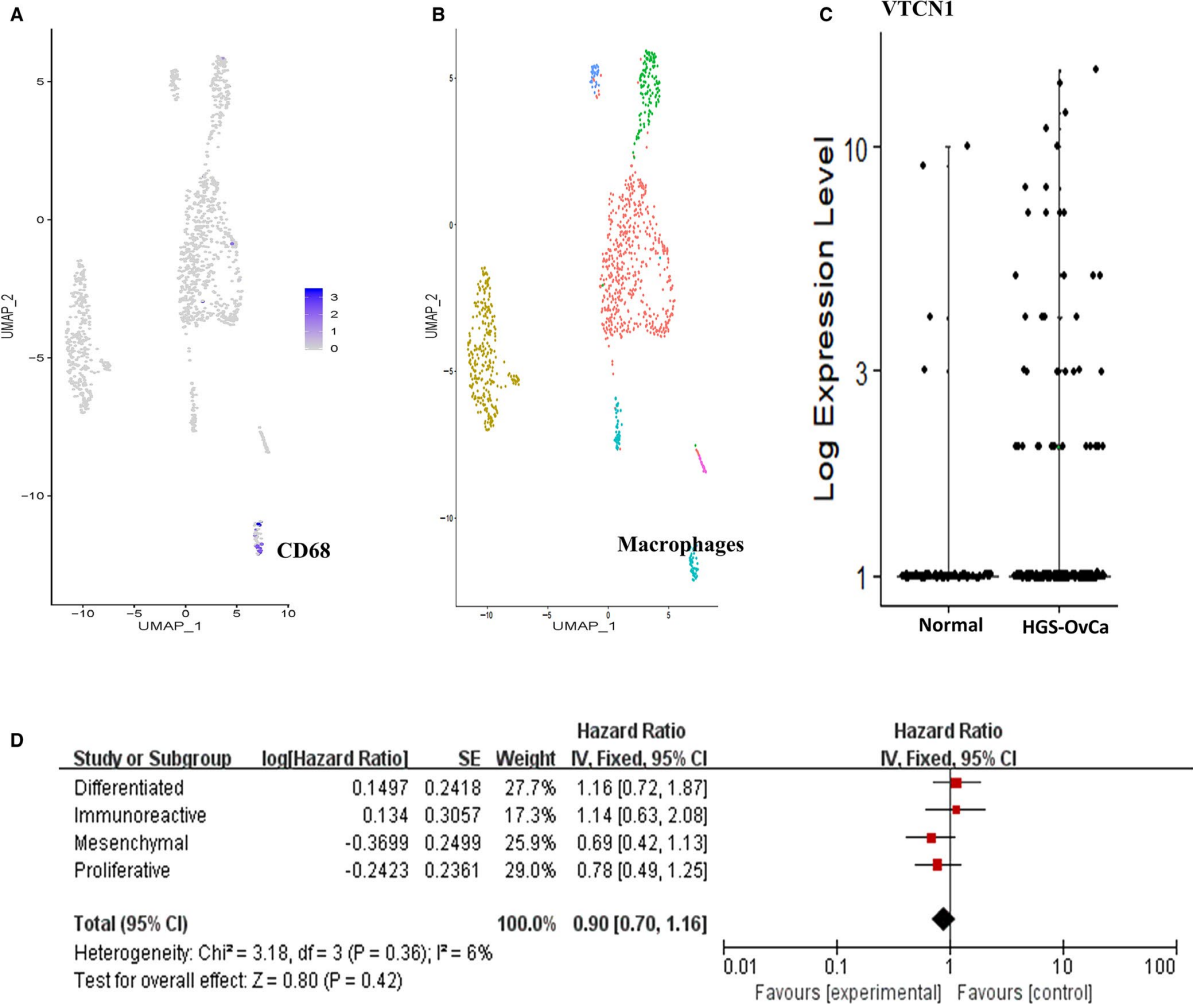
The possible origins and role of VTCN1 in immunoreactive HGS‐OvCa. t‐SNE plots for the GSE118828 dataset illustrate the cluster with high expression of CD68 (A) as macrophage (B). C, The comparison of the expression level of VTCN1 in macrophages between normal ovarian tissues and HGS‐OvCa tissues. D, The forest plot of the correlation between VTCN1 expression (vs. low expression) and the overall survival among the four subtypes of HGS‐OvCa patients

A previous study revealed that VTCN1 was mainly expressed in tumour‐associated macrophages, which had negative effects on the T‐cell response.^29^ Our analysis above found that immunoreactive HGS‐OvCa presents high abundant macrophage infiltration and up‐regulated expression of VTCN1. We analysed a published single‐cell RNA‐seq data set to explore the relationship between VTCN1 and tumour‐infiltrating macrophages. Macrophage signature CD68 was used to locate the macrophage cluster in the GESE118828 single‐cell data set (Figure 6A,B).^26^ The results confirmed that the VTCN1 is indeed mainly expressed in the tumour‐infiltrating macrophages (Figure 6C), but not in tumour cells. Finally, we reported the overall survival based on VTCN1 expression in each subgroup of HGS‐OvCa based on the TCGA cohort. It should be mentioned that although it did not reach the statistical significance, the high expression level of VTCN1 has the potential to be the risk factor (Figure 6D). This result suggests VTCN1 up‐regulated patients may have a distinct prognosis pattern compared with other patients.

## 4 DISCUSSION

HGS‐OvCa exhibits high levels of heterogeneity in its molecular and histological characteristics. The immunoreactive subgroup has a distinct immune cell‐infiltrated microenvironment and a generally favourable outcome.^30^ The aim of the present study was to gain a deeper understanding of the underlying mechanism and to identify potential therapeutic targets for HGS‐OvCa. We established a cross‐platform statistical classifier, which allows us to accurately classify 121 samples from 5 independent HGS‐OvCa gene expression dataset into immunoreactive and non‐immunoreactive cases. The classification of a large number of immunoreactive HGS‐OvCa provides sufficient power for us to identify true DEGs in immunoreactive HGS‐OvCa compared normal control samples. After removing genes also differentially up‐ and down‐regulated in general HGS‐OvCa samples.^28^ A total of 104 distinctly up‐regulated and 249 down‐regulated DEGs were identified as unique for immunoreactive HGS‐OvCa. Among these DEGs, immune‐related genes were mostly up‐regulated in immunoreactive HGS‐OvCa, while in general HGS‐OvCa, they were equally distributed among down‐regulated and up‐regulated genes.

KEGG analysis and GO analysis of the list of DEGs further revealed activation of the leukocyte TEM pathway in immunoreactive HGS‐OvCa, while the cell‐cycle‐related genes are commonly up‐regulated DEGs in general HGS‐OvCa and immunoreactive HGS‐OvCa. TEM refers to the process of leukocyte locomotion along with endothelial cells and across the border from the blood to the location of the inflammation. Thus, our analysis suggests that the phenotype of immunoreactive HGS‐OvCa is largely driven by immune cell infiltration. The PPI network analysis similarly identified significant module and hub genes related to immune response, and proteins significantly associated with the chemokine signalling pathway in immunoreactive HGS‐OvCa. Among them, proteins in Module 2 are significantly associated with the chemokine signalling pathway such as CXCL13 and CXCR4. CXCL13 serves as a chemoattractant that migration of B lymphocytes and chemotaxis of cells expressing CXCR5. CXCR4 is a chemokine receptor commonly expressed on most hematopoietic cell types including macrophages, monocytes, T and B lymphocytes, as well as ovarian cancer cells. Previous studies have shown that CXCL13 and CXCR4 expressions are associated with better prognosis in TP53 mutant ovarian cancer patients.^31^

Given that not merely the number of tumour‐infiltrating immune cells, but the type of infiltrating immune cells are important for proper immune surveillance and control of tumour cells, we applied the recently developed computation approach to quantify the abundance of infiltrating immune cells in HGS‐OvCa sample based on the bulk RNA‐seq and gene expression array data. We found the macrophages significantly enriched and infiltrated in the microenvironment compared with the other 3 subgroups and normal tissues based on the results from GSEA and TIMER analysis. The results could partly explain why the prognosis of this subtype is still relatively poor in HGS‐OvCa.^10^

Among the immune‐related genes overexpressed in immunoreactive HGS‐OvCa, we identified three candidate immunotherapy targets including *IDO1*, *CX3CL1* and *VTCN1*. IDO1 is the first key rate‐limiting enzyme in the tryptophan metabolic oxidation pathway.^32^ This enzyme is known as an ‘immune checkpoint’ because high expression could result in the suppression of the immune response and poor patient prognosis.^33^ IDO1‐mediating tumour immune escape is a result of two major effects of IDO1. First, the high expression level of IDO1 protein could lead to a reduction in Trp, and the lack of Trp inhibits the activation of responding T cells through the mTORC1 and GCN2 pathways.^34^ Second, the accumulation of the downstream kynurenines (Kyn) could promote the expression of IL‐10, followed by the transformation of *CD4*+ T cells to *FOCP3*‐expressing regulatory T cells, and eventually inhibit the normal immune response.^35^ Thus, although immune cells are recruited to the tumour microenvironment, IDO1 could suppress the immune response by inhibiting the responding T cells and converting the *CD4*+ T cells to regulatory T cells.

Several IDO1 inhibitors have been investigated in pre‐clinical analyses, and the data have shown that targeting IDO1 protein is safe and well‐tolerated. Although the response rate of single‐agent treatment of IDO1 is only 10%‐18%,^36^ combined chemotherapy or other immunotherapy could yield a promising objective response rate (ORR, up to 57%).^37^ A previous trial showed that the disease control rate (DCR) of the selective IDO1 inhibitor epacadostat plus pembrolizumab reached 35% (n = 13) in HGS‐OvCa patients and that the ORR was 8% (n = 3).^38^ The variations in ORR and DCR might be because of the complexity of the IDO1 mechanisms. Thus, given these results, IDO1 might be a very promising target for HGS‐OvCa.

VTCN1, also called B7‐H4, is a negative regulator of the T‐cell response and shows an 18% amino acid identity with human PD‐L1.^39^ This protein is mainly expressed on the cell surface of antigen‐presenting cells. Moreover, it can significantly inhibit TAA‐specific T‐cell proliferation and promote the proliferation of Treg cells. Macrophages are the most common antigen‐presenting cells in tumour stroma.^29^ Macrophages are classified as M1 and M2. M1 plays an antitumour role by producing IL12 and TNF‐a to induce the immune response, while M2 promotes tumour growth and angiogenesis by tumour promoters.^40^ A previous study suggested that macrophages expressed in the HGS‐OvCa microenvironment could activate B7‐H4 expression by autocrine mechanisms. B7‐H4+ macrophages spontaneously expressed IL10 and IL6 protein. Treg cells also produced high levels of IL6 and IL10 in ovarian tumours. These autocrine factors convert macrophages from the M1 stage to the M2 stage. The expression level of B7‐H4 protein in tumour‐associated macrophages is significantly related to the poor prognosis of HGS‐OvCa patients.^41^ Our results confirmed that VTCN1 was significantly up‐regulated in the macrophages in immunoreactive HGS‐OvCa (Figure 6).

Interestingly, *IDO1*, *VTCN1* and *CX3CL1* are protective factors in HGS‐OvCa, indicating that high expression levels of these factors are associated with prolonged survival time. However, we found that although it did not reach the statistical significance, the high expression level of *VTCN1* has the potential to be the risk factor.

In the present study, we built a cross‐platform HGS‐OvCa subgroup classifier and applied a set of integrated bioinformatic tools to systematically analyse the characteristics of immunoreactive HGS‐OvCa. We clarified that TEM and the chemokine signalling pathway were specifically involved in the formation of the tumour‐associated microenvironment. Moreover, macrophages showed significantly high expression levels in immunoreactive HGS‐OvCa either compared with normal tissue or other subtypes. Two immune checkpoints were up‐regulated in immunoreactive HGS‐OvCa. Their differential expression contributes to the distinct immunoreactive HGS‐OvCa tumour‐associated microenvironment.

## 5 CONCLUSIONS

In conclusion, the present study built an effective HGS‐OvCa subtype classifier to sort immunoreactive HGS‐OvCa samples in TCGA and GEO data sets. The unique molecular and immune characteristics of immunoreactive HGS‐OvCa are revealed. The immunoreactive HGS‐OvCa exhibit an aberrant activated chemokine signalling and the leukocyte TEM pathway. Its tumour‐associated microenvironment is main infiltrated by macrophages, rather than lymphocytes. Furthermore, VTCN1 might be the key gene to explain the immune resistance development of immunoreactive HGS‐OvCa.

## CONFLICT OF INTEREST

The authors declare that they have no competing interests.

## AUTHOR CONTRIBUTIONS

**Zheran Liu:** Data curation (equal); formal analysis (lead); investigation (equal); methodology (equal); resources (equal); software (equal); validation (equal); visualization (equal); writing‐original draft (lead); writing‐review and editing (equal). **Haifang Wu:** Data curation (equal); investigation (equal); methodology (equal); resources (equal); software (equal); writing‐review and editing (equal). **Jiachen Deng:** Data curation (equal); methodology (equal); project administration (equal); resources (equal); software (equal); visualization (equal); writing‐review and editing (equal). **Haoqing Wang:** Data curation (equal); formal analysis (equal); funding acquisition (equal); methodology (equal); validation (equal); writing‐review and editing (equal). **Zixuan Wang:** Data curation (equal); investigation (equal); validation (equal); visualization (equal); writing‐review and editing (equal). **Ailin Yang:** Data curation (equal); methodology (equal); resources (equal); software (equal); writing‐review and editing (equal). **Bowen Liang:** Software (equal); validation (equal); writing‐review and editing (equal). **Ji Luo:** Formal analysis (equal); methodology (equal); software (equal); validation (equal); visualization (equal); writing‐review and editing (equal). **Jianyong Li:** Data curation (equal); writing‐review and editing (equal). **Yanmei Xu:** Data curation (equal); formal analysis (equal); funding acquisition (equal); supervision (equal); writing‐review and editing (equal). **Xiaoli Tang:** Conceptualization (equal); supervision (equal). **Fen Fu:** Conceptualization (lead); funding acquisition (equal); methodology (equal); supervision (equal); writing‐review and editing (equal). **Li‐Bin Deng:** Conceptualization (equal); funding acquisition (equal); supervision (lead); validation (equal); visualization (equal); writing‐review and editing (lead).

## Supporting information

Supinfo 1

Supinfo 2

Supinfo 3

Supinfo 4

Supinfo 5

Supinfo 6

Supinfo 7

Supinfo 8

Supinfo 9

Supinfo 10

## Data Availability

The data that support the findings of this study are available from the corresponding author upon reasonable request.
