# Supplementary figures and images for: Molecular classification and immunologic characteristics of immunoreactive high‐grade serous ovarian cancer
Source: J Cell Mol Med. 2020 Jun 17;24(14):8103–14. doi: 10.1111/jcmm.15441 (PMC7348149; doi:10.1111/jcmm.15441)

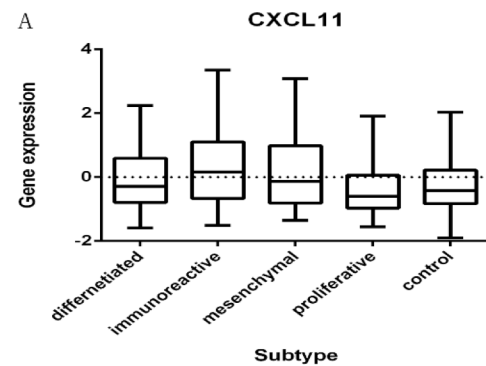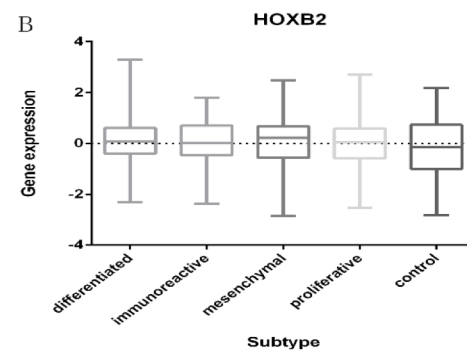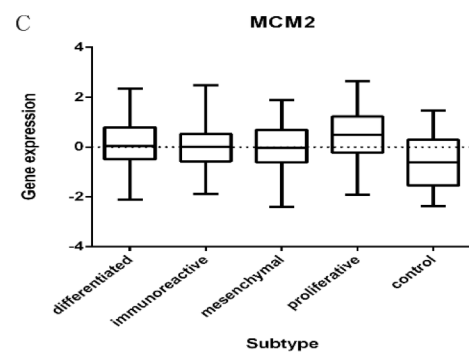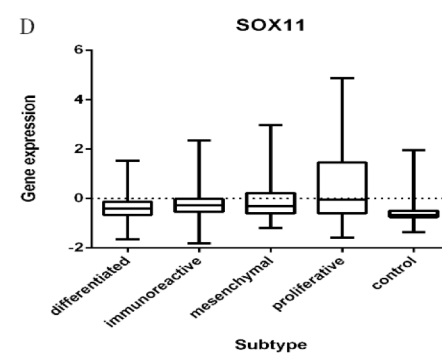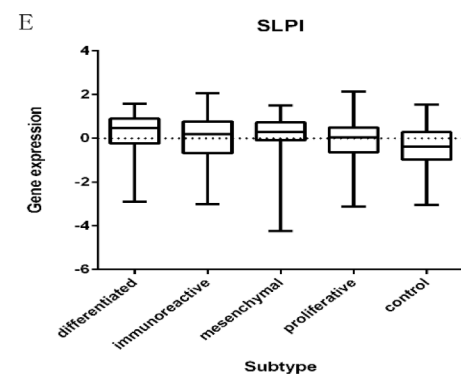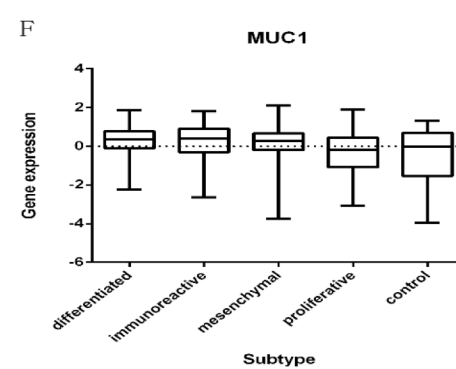

Supplement: Supplementary file 2 — Supinfo 2 [file JCMM-24-8103-s002.pdf]

## IDO1

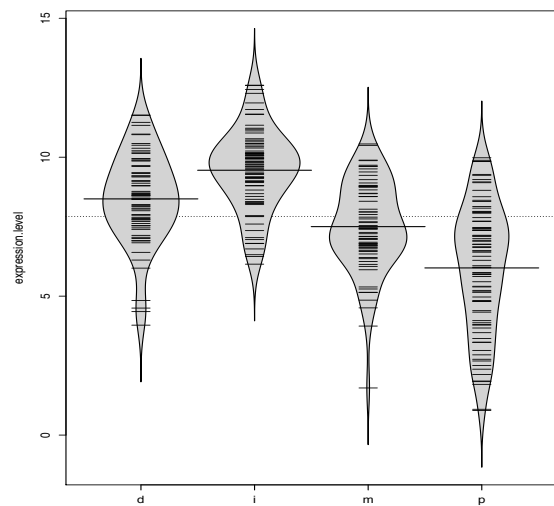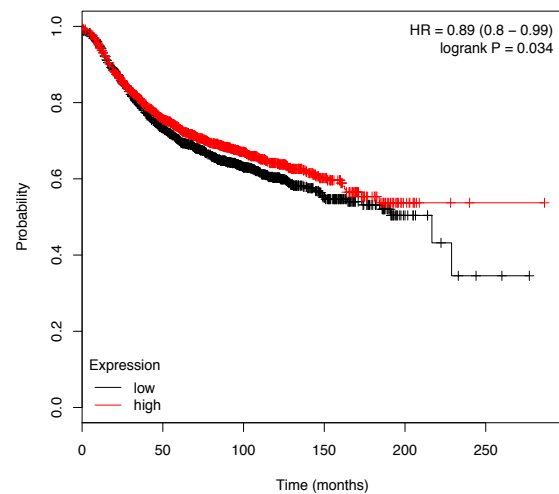

## VTCN1

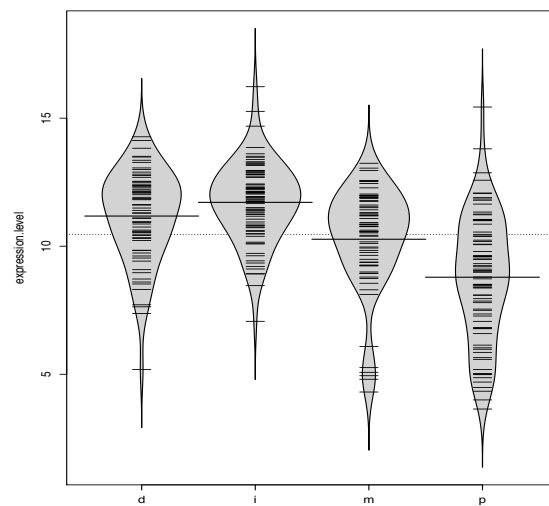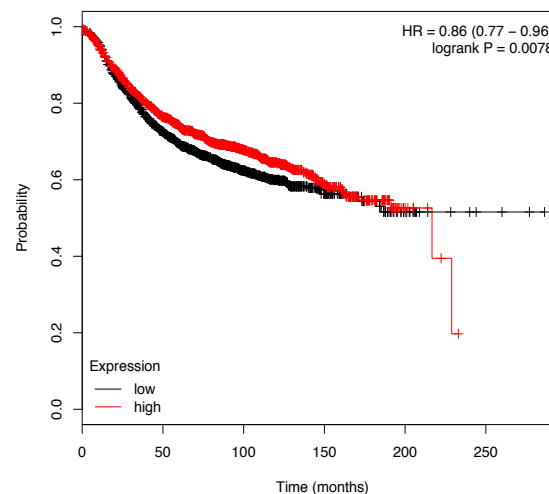

## CX3CL1

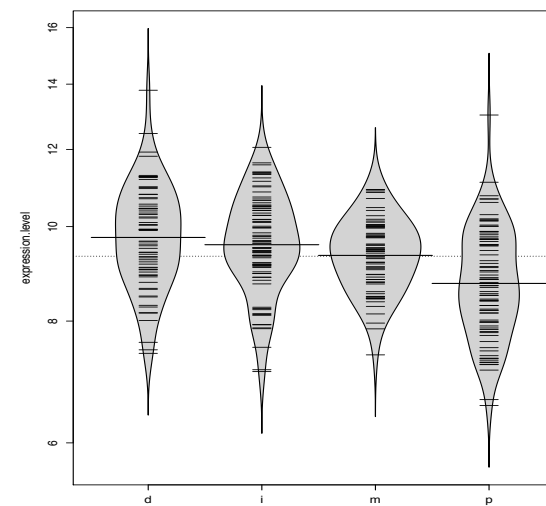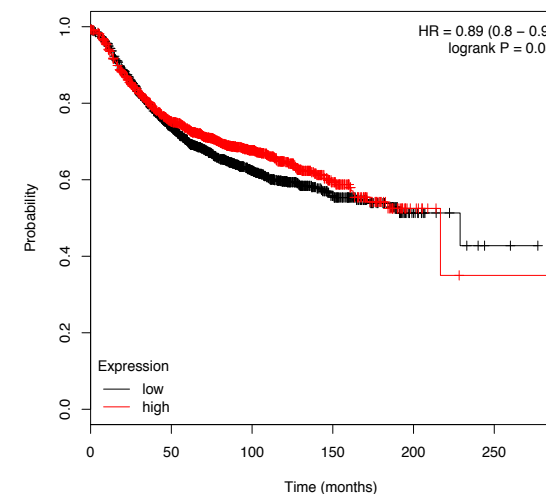

Supplement: Supplementary file 10 — Supinfo 10 [file JCMM-24-8103-s010.pdf]
