# Supplementary material for: Molecular classification and immunologic characteristics of immunoreactive high‐grade serous ovarian cancer
Source: J Cell Mol Med. 2020 Jun 17;24(14):8103–14. doi: 10.1111/jcmm.15441 (PMC7348149; doi:10.1111/jcmm.15441)
Supplement: Supplementary file 8 — Supinfo 8 [file JCMM-24-8103-s008.pdf]

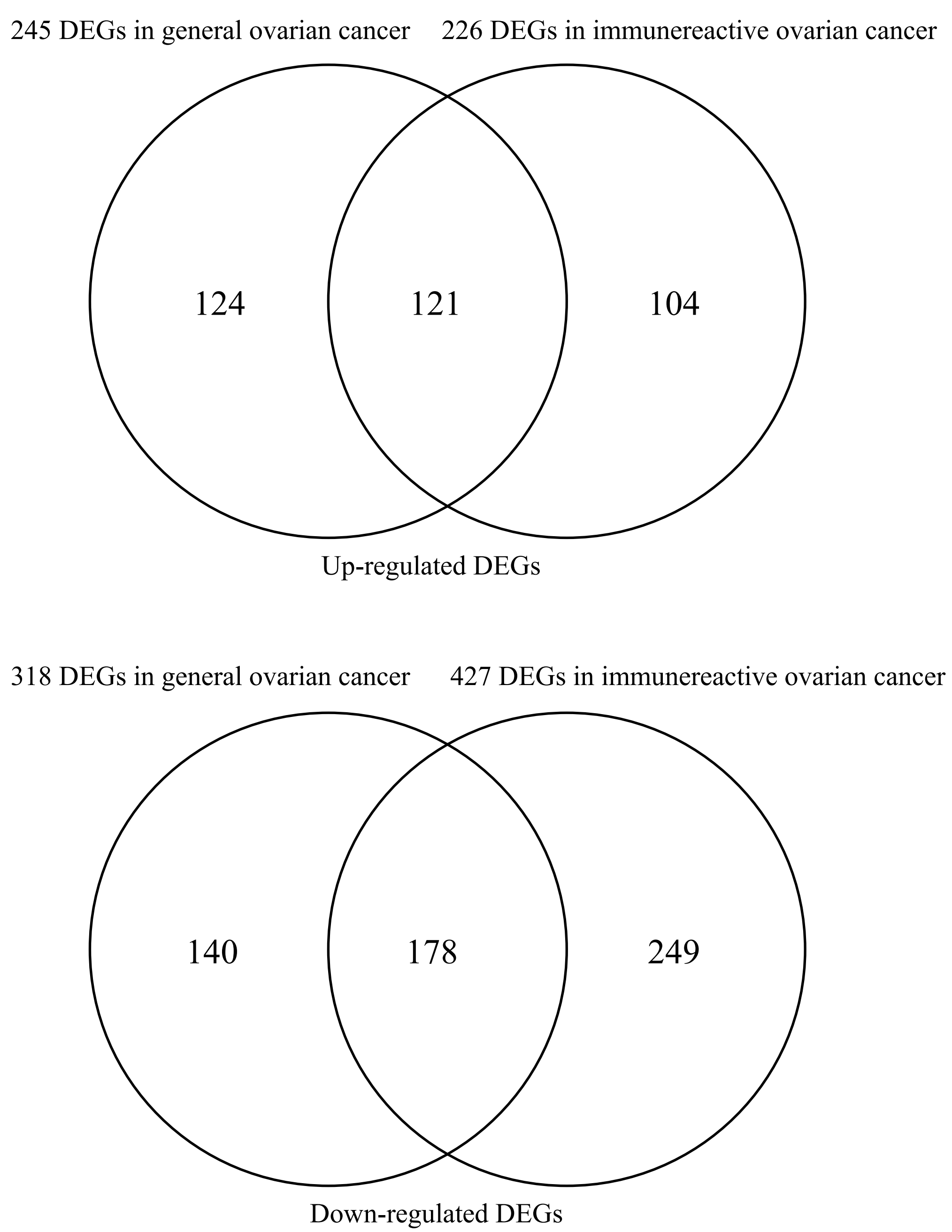
245 DEGs in general ovarian cancer
226 DEGs in immunereactive ovarian cancer
124
121
104
Up-regulated DEGs
318 DEGs in general ovarian cancer
427 DEGs in immunereactive ovarian cancer
140
178
249
Down-regulated DEGs
